# Integrated Smart Gas Tracking Device with Artificially Tailored Selectivity for Real-Time Monitoring Food Freshness

**DOI:** 10.3390/s23198109

**Published:** 2023-09-27

**Authors:** Yuli Xu, Zicheng Liu, Jingren Lin, Jintao Zhao, Nguyen Duc Hoa, Nguyen Van Hieu, Alexander A. Ganeev, Victoria Chuchina, Abolghasem Jouyban, Daxiang Cui, Ying Wang, Han Jin

**Affiliations:** 1 Institute of Micro-Nano Science and Technology, School of Electronic Information and Electrical Engineering, Shanghai Jiao Tong University, Shanghai 200240, China; yuki0612@sjtu.edu.cn (Y.X.); ryukosei@sjtu.edu.cn (Z.L.); linjingren@sjtu.edu.cn (J.L.); ljustyt01@sjtu.edu.cn (J.Z.); dxcui@sjtu.edu.cn (D.C.); 2 International Training Institute for Material Science, Hanoi University of Science and Technology, Hanoi 100000, Vietnam; hoa.nguyenduc@hust.edu.vn; 3 Faculty of Electrical and Electronic Engineering, Phenikaa University, Hanoi 100000, Vietnam; hieu.nguyenvan@phenikaa-uni.edu.vn; 4 Department of Chemistry, St Petersburg University, 7/9 Universitetskaya Emb., St. Petersburg 199034, Russia; ganeev@lumex.ru (A.A.G.); v.chuchina@spbu.ru (V.C.); 5 Pharmaceutical Analysis Research Center, Faculty of Pharmacy, Tabriz University of Medical Sciences, Tabriz 51368, Iran; ajouyban@hotmail.com; 6 National Engineering Research Center for Nanotechnology, Shanghai 200241, China; 7 Chengdu Environmental Investment Group Co., Ltd., Building 1, Tianfushijia, No. 1000 Jincheng Street, Chengdu 610000, China; 8 Department of Biological Science, College of Life Sciences, Sichuan Normal University, Chengdu 610101, China

**Keywords:** artificially tailored selectivity, smart gas sensor, yttria-stabilized zirconia (YSZ), volatile compounds, food freshness

## Abstract

The real-time monitoring of food freshness in refrigerators is of significant importance in detecting potential food spoiling and preventing serious health issues. One method that is commonly reported and has received substantial attention is the discrimination of food freshness via the tracking of volatile molecules. Nevertheless, the ambient environment of low temperature (normally below 4 °C) and high humidity (90% R.H.), as well as poor selectivity in sensing gas species remain the challenge. In this research, an integrated smart gas-tracking device is designed and fabricated. By applying pump voltage on the yttria-stabilized zirconia (YSZ) membrane, the oxygen concentration in the testing chamber can be manually tailored. Due to the working principle of the sensor following the mixed potential behavior, distinct differences in sensitivity and selectivity are observed for the sensor that operated at different oxygen concentrations. Typically, the sensor gives satisfactory selectivity to H_2_S, NH_3_, and C_2_H_5_OH at the oxygen concentrations of 10%, 30%, and 40%, respectively. In addition, an acceptable response/recovery rate (within 24 s) is also confirmed. Finally, a refrigerator prototype that includes the smart gas sensor is built, and satisfactory performance in discriminating food freshness status of fresh or semi-fresh is verified for the proposed refrigerator prototype. In conclusion, these aforementioned promising results suggest that the proposed integrated smart gas sensor could be a potential candidate for alarming food spoilage.

## 1. Introduction

The issue of food freshness has garnered significant attention due to its direct impact on human health and safety [1]. In the context of urban living, it is common practice to store various food items in a refrigerator and subsequently consume them over a specific duration. Nevertheless, it is important to note that different types of food have the potential to deteriorate over a period of time. This degradation may go unnoticed by individuals due to the relatively airtight conditions within a refrigerator, hindering their ability to detect any visual or olfactory signs of rotting. The occurrence of food spoilage in refrigerated storage facilities contributes significantly to the annual volume of food waste, while the consumption of such deteriorated food items can lead to the manifestation of severe health complications. Therefore, real-time monitoring of the freshness of food stored in the refrigerator is of great significance since it will be helpful to alarming the potential food spoilage, and thereby can effectively prevent severe health problems (e.g., food poisoning) that may be caused by eaten rotten food [2,3].

There are several elements that might influence the freshness of food, including microorganisms, temperature, humidity, and storage conditions. Hence, the assessment of food freshness within the refrigerator is a highly intricate process [4]. The microbiological and chemical processes, like changes in protein and lipid fractions, are mainly responsible for the spoilage of fresh food [5]. Up to date, methods that have been frequently reported for food freshness monitoring include but are not limited to microbiological testing, visual recognition, capillary electrochromatography, volatile compounds analysis, etc. [6,7,8,9,10,11]. Among them, discriminating food freshness by tracking volatile compounds receives extensive attention due to its attractive characteristics of rapid and non-invasive, which are suitable for alarming food spoilage at an early stage [12,13,14]. Traditionally, the analysis of volatile substances has been conducted using several methods, such as gas chromatography (GC) [15], ion mobility spectrometry (IMS) [8], and proton-transfer-reaction mass spectrometry (PTR-MS) [16]. These methodologies offer both qualitative and quantitative assessments of volatile substances. However, they necessitate the involvement of skilled individuals and entail significant operational expenses [17]. Additionally, the sample preparation and analysis procedures associated with these approaches are time-consuming. In addition, it should be noted that these devices possess a substantial size and necessitate regular calibration [18,19,20]. This has motivated significant efforts for the development of gas sensors that may offer a more rapid, user-friendly, miniaturized, non-destructive, and affordable detection of several important gases with high selectivity and sensitivity [21]. Until present, high-performance silicon transistor arrays for trace-level, multi-gas sensing have effectively assessed food freshness in complex gaseous environments for practical applications [13]. However, in consideration of the ambient environment of low temperature (normally below 4 °C) and high humidity (90% R.H.) in refrigerators, it is highly desired to develop high-performance chemical gas sensors when tacking target volatile gas species [7,9,14,22,23,24].

When food is refrigerated, some components disintegrate and emit odors as a result of the reaction of bacteria and enzymes. Proteins undergo degradation, resulting in the formation of spoilage amines, which then break down into ammonia, hydrogen sulfide, and ethyl mercaptan [25]. The process of fat decomposition involves the breakdown of fat molecules into fatty acids, which are subsequently converted into aldehydes [26]. Carbohydrates undergo decomposition processes that result in the formation of alcohols, ketones, and aldehydes [27]. Therefore, to effectively monitor food freshness, three main target volatile compounds should be particularly focused on, namely, H_2_S (derived from meat spoilage), NH_3_ (derived from meat spoilage), and C_2_H_5_OH (derived from vegetable/fruit spoilage) [1,28]. In comparison with chemiresistive-type gas sensors, solid-state yttria-stabilized zirconia (YSZ)-based gas sensors demonstrate reliable performance in harsh conditions [4,29,30,31,32,33,34]. In addition, because of the relatively high selectivity, it is expected that YSZ-based gas sensors could be a promising candidate for sensing these volatile compounds in refrigerators [35,36,37,38]. Nevertheless, highly selective tracking of the aforementioned H_2_S, NH_3_, and C_2_H_5_OH against other potential co-existing interference gases, like CO, NO, NO_2_, and SO_2_, remains a challenge [39,40,41].

It is widely recognized that gas sensors based on yttria-stabilized zirconia (YSZ) typically exhibit mixed potential behavior when exposed to gas mixtures containing oxygen [39,42,43,44]. Consequently, the sensing characteristics (i.e., sensitivity and selectivity) of this type of sensor could be manipulated artificially by varying the oxygen concentration in the gas mixture [42,45]. In consequence, this research proposes an integrated smart gas sensor with artificially tailored selectivity for monitoring volatile compounds typically generated during food deterioration. Using a YSZ membrane to control the oxygen concentration in the testing chamber, an integrated ceramic-based oxygen pump is created so that the selectivity of a miniaturized YSZ-based electrochemical gas sensor can be readily adjusted. In the interim, the practicability of the integrated smart gas sensor to monitor the level of food preservation in the refrigerator will be systematically assessed. This research is believed to provide an alternative strategy for designing high-performance gas sensors for future food quality evaluation applications.

## 2. Experimental Section

### 2.1. Materials Preparation

Materials (e.g., SnO_2_, Co_3_O_4_, ZnO, NiO, Cr_2_O_3_, and In_2_O_3_) with sensing behavior that is widely reported to be easily affected by oxygen concentration were selected as research objectives [46,47,48,49]. All the materials are directly bought from Sigma-Aldrich Chemie GmbH, Taufkirchen, Germany.

### 2.2. Fabrication of the Integrated Smart Gas Sensor

**Figure 1:**
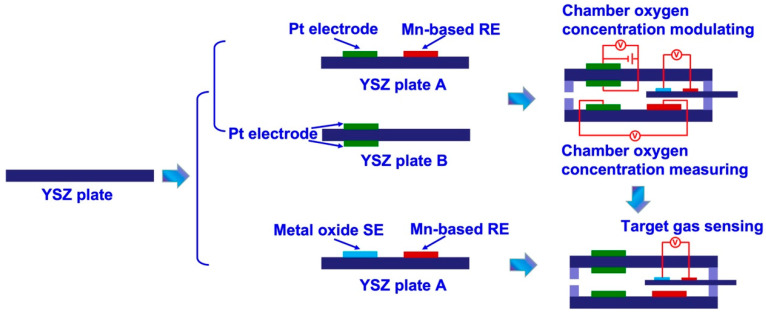
Schematical view of the details of fabricating the integrated smart gas sensors.

The intelligent gas sensor is composed of two parts, namely, a testing chamber (chamber oxygen concentration controlling module) and a miniaturized YSZ-based sensor (target gas sensing module). The fabrication details of each part are summarized as follows:

(i) Fabrication of the chamber oxygen concentration controlling module: Initially, Pt paste (TANKA Precious Metals, Tokyo, Japan) and Mn_2_O_3_ paste were painted on the surface of commercial YSZ plates (length × width × thickness: 1.75 × 0.5 × 0.1 cm^3^; NIKKATO CORPORATION, Osaka, Japan) to form the oxygen pumping and sensing electrode. After that, two pieces of the as-fabricated commercial YSZ plates were bound together with the help of glass cement, followed by calcined at 1400 °C for 2.5 h to form the chamber oxygen concentration controlling module. The space of the internal testing chamber is about 0.06 cm^3^ (length × width × thickness: 1.5 × 0.4 × 0.1 cm^3^, as shown in Figure 1).

(ii) Fabrication of the target gas sensing module and integrated smart gas sensor: YSZ-based sensors are fabricated using commercial YSZ plates (length × width × thickness: 2 × 0.3 × 0.1 cm^3^; NIKKATO CORPORATION, Osaka, Japan). To simplify the configuration of the fabricated sensors, manganese-based reference electrode (hereafter denoted as Mn-based RE) was used on these sensors. A commercially available Mn_3_O_4_ powder (99% purity, Sigma Aldrich Chemie GmbH, Taufkirchen, Germany) was meticulously blended with α-terpineol. The resulting paste was subsequently applied onto the surface of YSZ plates, resulting in the formation of a 1 mm Mn_3_O_4_-banded electrode. After undergoing a drying process at a temperature of 130 °C for an entire night, YSZ plates that contained a layer of Mn_3_O_4_ were subjected to calcination at a temperature of 1400 °C for 2.5 h in an environment consisting of air. This calcination process aimed to facilitate the formation of the Mn-based RE. Each of the sensing materials, namely SnO_2_, Co_3_O_4_, ZnO, NiO, Cr_2_O_3_, and In_2_O_3_ (purchased from Sigma Aldrich Chemie GmbH, Taufkirchen, Germany, with a purity of 99%), which were based on metal oxides, were individually applied onto the surface of the YSZ plates. This application was carried out to create an oxide layer that possessed similar dimensions to the Mn-based RE. Subsequently, each of the fabricated oxide layers underwent calcination at high temperatures to obtain the photoactive SEs. The calcination temperature for the Cr_2_O_3_-SE sensor ranged from 900 to 1100 °C, while for the sensor composed of the other SEs, the calcination temperature was fixed at 1000 °C. Subsequently, the YSZ-based sensors, in their as-fabricated state, were carefully placed within the testing chamber and securely sealed using glass cement, resulting in the creation of integrated smart gas sensors. Figure 1 displays a schematic representation of the sensor.

### 2.3. Evaluating the Gas Sensing Performance of the Integrated Smart Gas Sensor

Sensing experiments were performed by simultaneously exposing the sensors to the base gas (diluted 21 vol% O_2_ + N_2_ balance) or the sample gas containing each of different volatile compounds in the range of 0.04–2 ppm for each in the based gas to evaluate gas-sensing characteristics. Since H_2_S, NH_3_, and C_2_H_5_OH are the most prevalent volatile compounds emitted during food decomposition, these gas species were chosen as the sample gas for this investigation. The humidity and the temperature of the gas mixture are kept at 90% RH and 4 °C to simulate the operating condition in the refrigerator. To eliminate the negative impact derived from humidity and low ambient temperature, the sensor is operated at 425 °C on an internal aluminum oxide heating plate.

**Figure 2:**
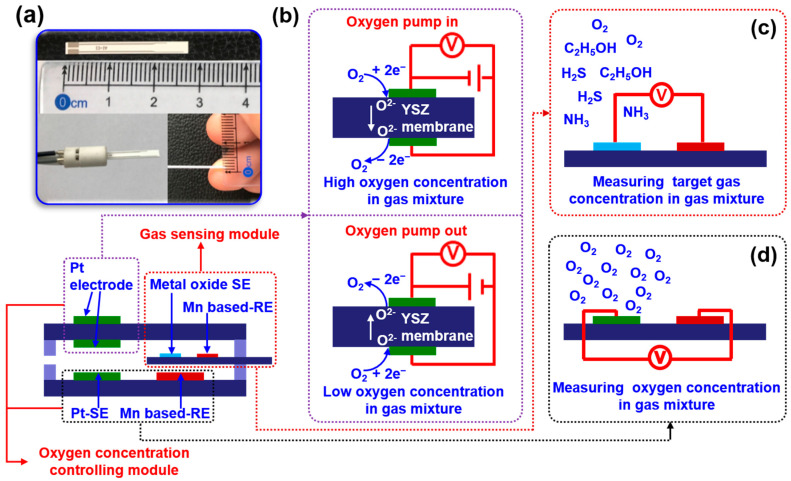
Illustration of the integrated smart gas sensor: (**a**) a miniaturized YSZ-based sensor; (**b**) oxygen pump principle; (**c**) manually controlling the oxygen concentration in the testing chamber; (**d**) measuring target gas concentration.

The oxygen concentration inside the testing chamber is manually modulated by applying pumping voltage on the pumping electrode, which is located on the surface of the YSZ membrane (Figure 2b). By controlling the pumping time, oxygen concentration in the testing chamber can be artificially fixed in the range of 10–40%. The sensing signal of electric potential difference Δ*V* (Δ*V* = *V*_sample gas_ − *V*_base gas_, where the *V*_sample gas_ represents the sensing response of the sensor towards sample gas, whereas the *V*_base gas_ represents the sensing response of the sensor towards base gas) was recorded using a multi-function data acquisition board (USB-6211, National Instruments, Austin, TX, USA). The 90% response/recovery time was calculated in the following way: the maximum value of the sensor given at response/recovery within the examined time was denoted as the Δ*V*_response/recovery_, and then, the value of 90% Δ*V*_response/recovery_ can be read from the figure, and its corresponding time was defined as the 90% response/recovery time.

When operating the sensor within the refrigerator prototype, oxygen concentration in the testing chamber would be tailored to the set value within 5 s for each measurement. After that response signal of the sensor will be calculated with the equation of Δ*V* = *V*_sample gas_ − *V*_base gas_, in which the Vsample gas represents the sensing response of the sensor towards sample gas derived from refrigerator.

The current–voltage (polarization) curves were measured with an electrochemical analyzer system (CHI604D, Chenhua, China) by employing the linear potential-sweep method at a scan rate of 0.1 mV/s at 475 °C in air base (or sample gas). In brevity, the standard modified polarization curve for the cathodic reaction of air was obtained by plotting the applied potential against the absolute current value. The modified polarization curve for the anodic reaction of the sample gas was estimated by subtracting the current value in the base gas from the current value in the sample gas at each potential.

## 3. Results and Discussion

### 3.1. General Vision of the Integrated Smart Gas Sensor

An integrated smart sensor is proposed and designed for food quality monitoring. The proposed sensor is composed of a chamber oxygen concentration controlling module and a target gas sensing module (Figure 2a). When exposing the sensor to the gas mixture, the oxygen concentration in the gas mixture will be automatically modulated with the help of the oxygen concentration controlling module. By applying pumping voltage on the YSZ membrane, oxygen can be controllable and pumped out of or inside the testing chamber, leading to low or high oxygen concentration in the gas mixture (in the testing chamber), as shown in Figure 2b. A gas sensor based on yttria-stabilized zirconia (YSZ) will be developed in a downsized form and integrated into the testing chamber. This sensor aims to achieve superior performance in detecting certain gases, as seen in Figure 2c. Meanwhile, the oxygen concentration within the testing chamber is continuously monitored in real time to manually adjust it to the desired value. Due to the YSZ-based sensor following the working principle of mixed potential behavior, its sensing characteristics will directly be tailored via the oxygen concentration in the gas mixture (in the testing chamber). Consequently, high selectivity to specific gas at certain oxygen concentrations is expected.

### 3.2. Sensing Performance of the Proposed Smart Gas Sensor

**Figure 3:**
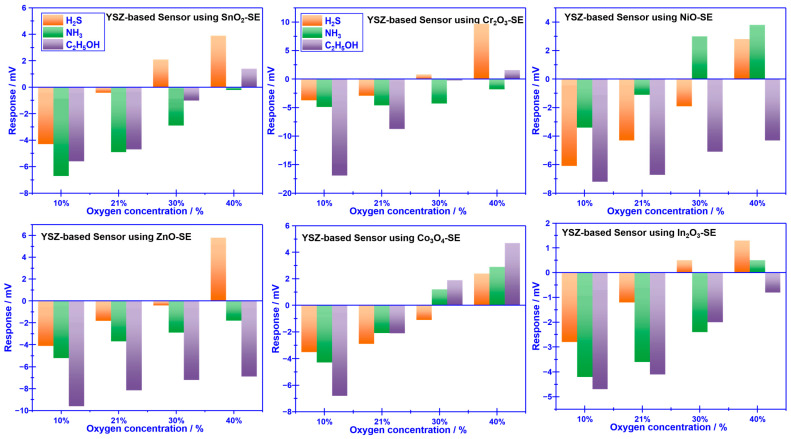
Sensing characteristics of the smart gas sensor attached with various SEs and Mn-based Res under different oxygen concentrations.

To realize the aforementioned attractive vision, the sensing behavior of YSZ-based gas sensors using a variety of commonly reported sensing materials (e.g., SnO_2_, Co_3_O_4_, ZnO, NiO, Cr_2_O_3,_ and In_2_O_3_) as the SE (vs. Mn-based RE) to 0.04 ppm H_2_S, NH_3_, and C_2_H_5_OH was initially characterized under different oxygen concentrations (10%, 21%, 30%, and 40%). As depicted in Figure 3, the response signal of the YSZ-based gas sensors attached with various sensing materials (as the sensing electrode, SE) vs. the Mn-based reference electrode (RE) varied with the oxygen concentration, and most of these sensors exhibited unexpected selectivity towards the studied gases. Nevertheless, it is intriguing to note that the sensor using Cr_2_O_3_-SE (vs. Mn-based RE) demonstrated relatively high selectivity to H_2_S, NH_3_, and C_2_H_5_OH at various oxygen concentrations. For instance, the response signal of the sensor to 0.04 ppm H_2_S, NH_3_, and C_2_H_5_OH under the oxygen concentration of 10%, 30%, and 40% is (−3.7 mV, −4.9 mV, and −17.3 mV), (0.8 mV, −4.3 mV, and −0.2 mV) and (8.7 mV, −1 mV, and 1.6 mV), respectively. This encouraging result suggests that it is quite possible to achieve the desired high selectivity for detecting volatile compounds derived from spoiled food by modifying the oxygen concentration in the testing atmosphere.

**Figure 4:**
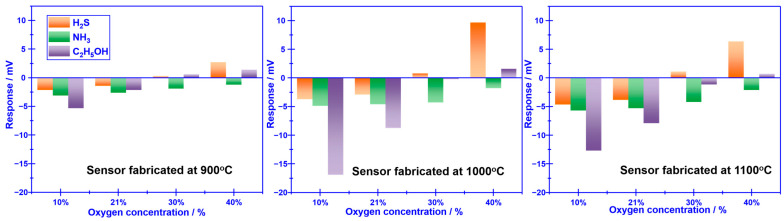
Impact of the calcination temperature on the sensing performance of the sensor attached with Cr_2_O_3_-SE.

The calcination temperature of the sensor attached with Cr_2_O_3_-SE is further optimized in the range of 900–1100 °C, with intervals of 100 °C (shown in Figure 4). The largest response signal and optimal selectivity are obtained for the sensor calcined at 1000 °C. The impact of calcination temperature can be explained via the balance of forming triple-phase boundary (TPB) and catalytic activity, which has been systematically discussed elsewhere [50]. TPB can be hardly formed at low calcination, leading to feeble electrocatalytic activity and low reactivity at the reaction interface (i.e., the interface between SE and YSZ). Even so, an excessively high calcination temperature normally destroys the catalytic activity of the sensing materials. As a result, a moderate calcination temperature not only aids in enhancing the interfacial reactivity by forming a desirable TPB but also maintains an adequate level of catalytic activity. Thus, optimal sensing performance is observed at the calcination temperature of 1000 °C for the YSZ-based sensor that is associated with Cr_2_O_3_-SE.

**Figure 5:**
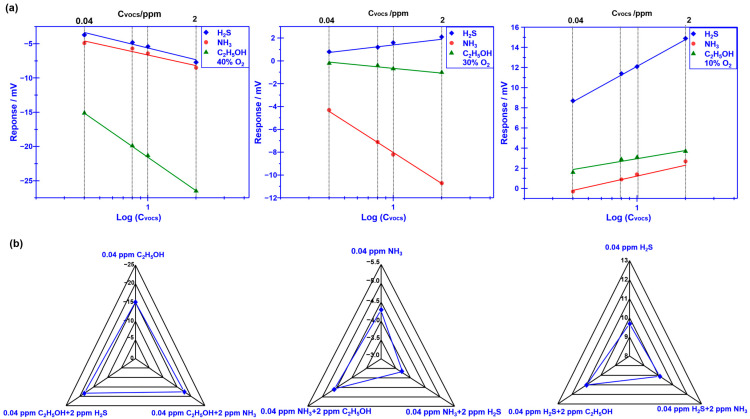
(**a**) Dependence of the response signal on the concentration of the studied gases for the sensor using Cr_2_O_3_-SE, in the range of 0.04–2 ppm is examined at different oxygen concentrations; (**b**) dependence of the response signal on the concentration of the studied gases for the sensor using Cr_2_O_3_-SE, in the range of 0.04–2 ppm is examined at different oxygen concentration. (**b**) Comparison of the response signal for the smart gas sensor to the studied gas mixture.

**Figure 6:**
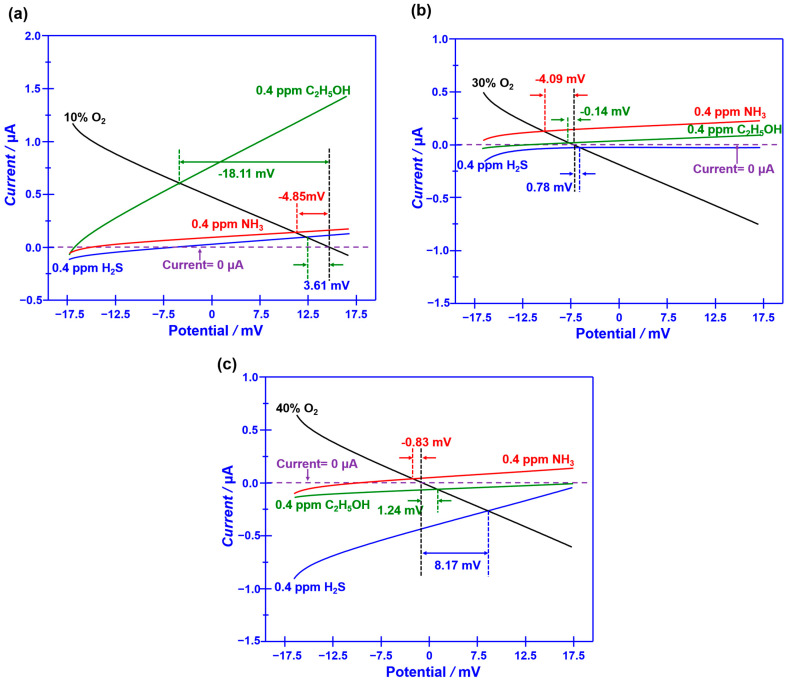
(**a**–**c**) Standard modified polarization curves, measured in 0.04 ppm H_2_S, NH_3_, and C_2_H_5_OH under different oxygen concentration values over the range of 10–40 vol.% (with intervals of around 10 vol.%), for the smart gas sensor coupled with Cr_2_O_3_-SE and Mn-based RE.

Figure 5a demonstrates the dependence of the response signal on the concentration of the studied gases for the YSZ-based sensor using Cr_2_O_3_-SE (calcined at 1000 °C) in the range of 0.04–2 ppm, which was examined at different oxygen concentrations. The linear relationship between the response signal and the logarithm of the gas concentration is observed for the sensor, regardless of the oxygen concentration contained in the gas mixture. In particular, acceptable selectivity is also confirmed for the sensor by modulating the oxygen concentration. To further confirm the gas sensing specificity, a comparison of the response signal to the 0.04 ppm single gas (e.g., H_2_S, NH_3_, and C_2_H_5_OH) and the gas mixture (target gas + 2 ppm interference gases) is investigated and shown in Figure 5b. Acceptable specificity is confirmed for the sensor when sensing various targeted gases under different oxygen content. These impressive results directly indicate that the selectivity of the proposed YSZ-based sensor that uses Cr_2_O_3_-SE is successfully tailored by simply modulating oxygen content in the tested environment. As for the tailored selectivity of the sensor, it can be interpreted via the standard modified polarization curves that are given in Figure 6. The difference in the modified polarization curves for each studied target gas at the oxygen concentration of 10–40% suggests that the interfacial catalytic activity of the sensor changed with the variation in the oxygen concentration. In particular, due to the mixed potential behavior of the sensor, distinct different sensing behavior is obtained when modulating the oxygen concentration in the gas mixture.

**Figure 7:**
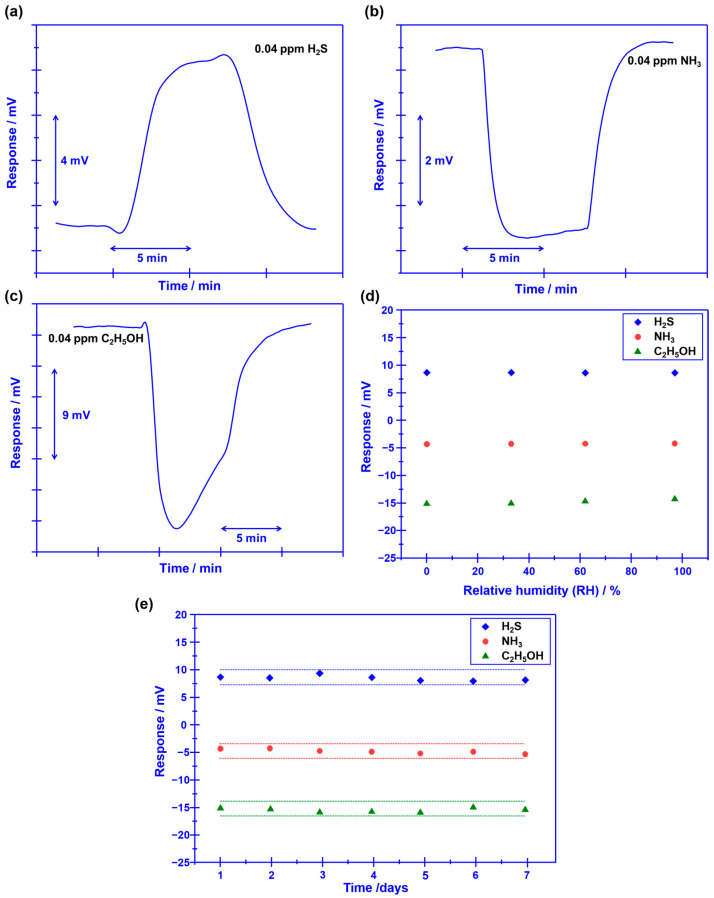
(**a**–**c**) A 90% response/recovery time of the sensor that uses Cr_2_O_3_-SE to H_2_S, NH_3_, and C_2_H_5_OH. (**d**) Impact of water vapor on the response behavior of the sensor using Cr_2_O_3_-SE. (**e**) Stability of the sensor to various gases within 1 week.

Beyond the satisfactory selectivity, quick response/recovery rate, and humidity resistance are also crucial technical indexes since the research objective of this study is tracking target volatile compounds that are generated during food spoilage. Figure 7a–c shows the response/recovery state, the 90% response/recovery time of the sensor to H_2_S, NH_3_, and C_2_H_5_OH is 23.6 s/17.1 s, 7.3 s/11.2 s, and 5.4 s/15.2 s, respectively, indicating acceptable response/recovery speed for sensing these gases. Meanwhile, water vapor has a negligible impact on the response behavior of the sensor, and acceptable stability is confirmed for the sensor (Figure 7d,e). Moreover, the noise of the sensor for each measurement is roughly estimated to be within 1 mV. Based on these promising results, it is reasonable to give the conclusion: the smart sensor that integrated oxygen pump membrane and miniaturized YSZ-based gas sensor (attached with Cr_2_O_3_-SE and Mn-based RE) shows excellent oxygen concentration modulation capability and satisfactory gas sensing characteristics, thus, could be a potential candidate for food freshness evaluation.

### 3.3. Capability of Real-Time Monitoring of Food Freshness

**Figure 8:**
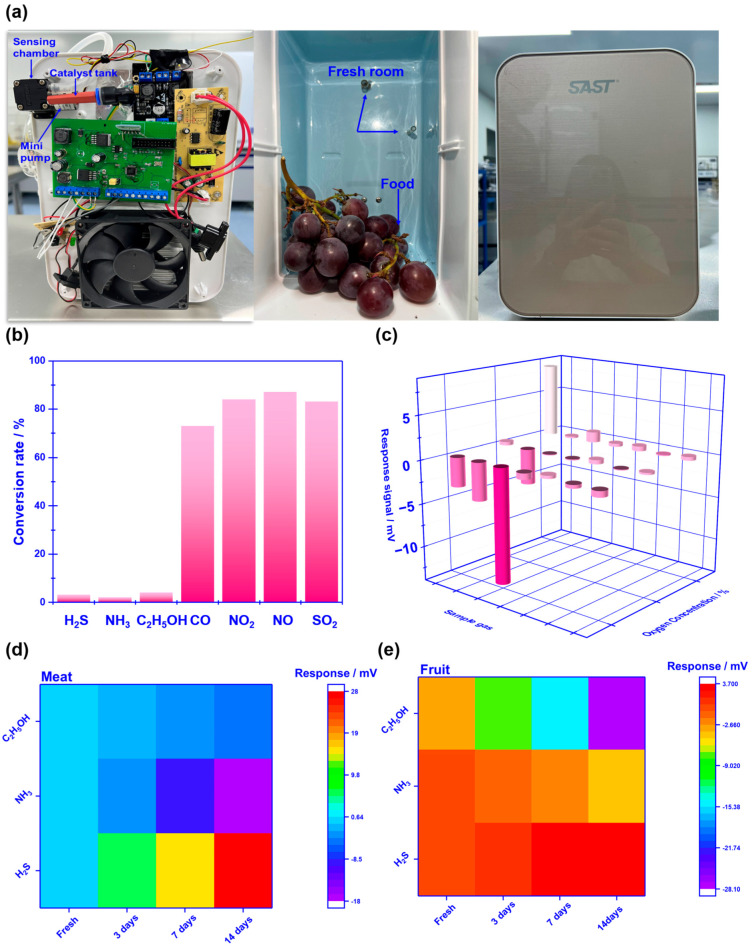
Photograph and sensing performance of the refrigerator prototype: (**a**) internal details of the refrigerator prototype; (**b**) comparison of the conversion rate for the studied gases before and after passing through the catalyst; (**c**) response signal of the integrated smart gas sensor against potential interference gases with the help of the commercialized catalyst; (**d**,**e**) variation in the response signal to H_2_S, NH_3_, and C_2_H_5_OH in the form of heat map during the studied 14 days.

To evaluate the practicability of employing the proposed sensor in freshness monitoring, a refrigerator prototype that includes the smart gas sensor is built, and its performance is also examined. It should be particularly noted that to effectively eliminate potential interference derived from other gases (e.g., CO, NO_2_, NO, and SO_2_), and a commercialized catalyst powder (C21900, Minstrong Technology CO., LTD., Changsha, China) is placed in front of the sensing chamber to remove potential interference gases. Figure 8a demonstrates the photograph of the designed prototype. The prototype includes a sensing chamber (placing the smart gas sensor), min-pump (sucking in gas derived from food), catalyst tank (placing commercialized catalyst powder), and other refrigerator modules. When the prototype works, gas species in a fresh room would be automatically pumped into the sensing chamber and examined via the sensor. Figure 8b gives the conversion rate of each gas, calculated by comparing the concentration difference before and after the gas passing through the catalyst. The catalyst essentially removed those potential interference gases due to the high conversion rate to CO, NO_2_, NO, and SO_2_. Therefore, the smart gas sensor placed behind the catalyst tank reveals a high response signal to H_2_S, NH_3_, and C_2_H_5_OH against the aforementioned interference gases (Figure 8c). Consequently, it is believed that the designed prototype would be capable of alarming food spoilage by simply tracking volatile compounds.

**Figure 9:**
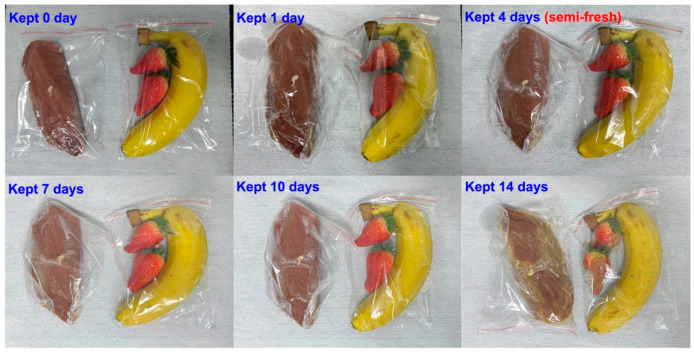
Photograph of pork and selected fruits that were kept for different times.

**Figure 10:**
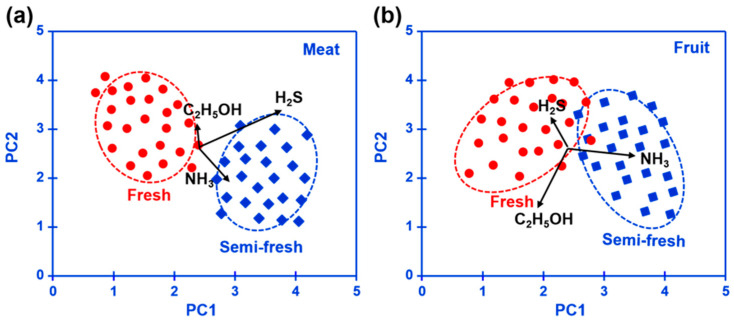
Pilot results of the (**a**) meat and (**b**) fruit freshness monitoring for the designed refrigerator prototype depicted in the form of a PCA map.

Pork, banana, and strawberry are selected as the research examples of meat and fruit to test the performance of the prototype. Fresh pork, strawberries, and bananas, weighing approximately 100–200 g, were procured from a local market in Shanghai. The perishable food items were expeditiously delivered to the laboratory using polystyrene containers that were filled with ice. Fresh goods were housed in a designated fresh room, where the temperature was maintained at 4 °C for 0–14 days for the purpose of preservation. Three replicates were prepared for each sample. The variation in the response signal to the gases sucked from the fresh room is continuously recorded during the tested period. It is found that when keeping these foods in a fresh room for more than 7 days, obvious color change can be caught in the photograph (Figure 9), suggesting the food spoilage started. Figure 8c, d shows a variation in the response signal to H_2_S, NH_3_, and C_2_H_5_OH in the form of a heat map during the studied 14 days. In sum, according to the variation in the response signal, it can be deduced that significantly increasing H_2_S produces NH_3_ found during meat spoilage, while for fruit spoilage, the gas concentration increment is mainly found in C_2_H_5_OH. Figure 10 summarizes the pilot results of freshness monitoring by utilizing the designed refrigerator prototype. In this research, the principal component analysis (PCA) method is employed to discriminate the status of fresh (kept within half a day) or semi-fresh (kept more than 3.5 days). Features of sensing response (each data repeated 3 times, as shown in Figure 8d,e) to these gases given via the sensor are recorded to create 12 × 3 (row × column) feature vectors as the input to PCA. Each row represents a single measurement, and each column represents a feature calculated for that particular measurement. The response signal of H_2_S and NH_3_ for the meat kept for 3.5 days is around 11.2 mV and −7.4 mV, respectively, while the value of C_2_H_5_OH for fruit at the semi-fresh status is roughly estimated to be −19.4 mV. These results depicted in the form of a PCA map imply the satisfactory capability of the prototype to distinguish food freshness, particularly when the experimental sample is meat. Conclusively, the prototype that integrated with the smart gas sensor successfully implemented online freshness monitoring and would provide an effective strategy for alarming food spoilage.

## 4. Conclusions

An integrated smart gas sensor is designed and fabricated for real-time monitoring of food freshness. By applying the pumping voltage on the YSZ membrane, the oxygen concentration in the testing chamber is artificially controlled. Due to the mixed potential behavior, the YSZ-based smart gas sensor that is affixed to Cr_2_O_3_-SE and Mn-based RE exhibits distinct different sensing characteristics at different oxygen concentrations. The sensor exhibits satisfactory selectivity for H_2_S, NH_3_, and C_2_H_5_OH at oxygen concentrations of 10%, 30%, and 40%, respectively, in the gas mixture. In addition, an acceptable response/recovery rate is further confirmed for the sensor.

To verify the capability of monitoring the food freshness for the proposed integrated smart gas sensor, a refrigerator prototype that includes the smart gas sensor is built. With the help of commercialized catalysts, high selectivity to these target gases (i.e., H_2_S, NH_3,_ and C_2_H_5_OH) that are emitted during food spoilage. Moreover, satisfactory performance in discriminating food freshness status of fresh or semi-fresh is further confirmed for the proposed refrigerator prototype. Conclusively, these promising results suggest that the proposed integrated smart gas sensor could be a potential candidate for alarming food spoilage. In particular, the strategy of modulating response selectivity by tailoring the oxygen concentration would be a useful way to design future high-performance gas sensors. However, it should be noted that the long-term stability and response frequency of the sensor still need to be further examined, which will be thoroughly studied in our future work.

## Data Availability

The data presented in this study are available on request from the corresponding author. The data are not publicly available due to the application of patents.
